# Acknowledgment to Reviewers of *Neurology International* in 2021

**DOI:** 10.3390/neurolint14010013

**Published:** 2022-01-25

**Authors:** Neurology International Editorial Office

**Affiliations:** MDPI AG, St. Alban-Anlage 66, 4052 Basel, Switzerland

Rigorous peer-reviews are the basis of high-quality academic publishing. Thanks to the great efforts of our reviewers, *Neurology International* was able to maintain its standards for the high quality of its published papers. Thanks to the contribution of our reviewers, in 2021, the median time to first decision was 29 days and the median time to publication was 57 days. The editors would like to extend their gratitude and recognition to the following reviewers for their precious time and dedication, regardless of whether the papers they reviewed were finally published:
Aaron LernerJakov MilićAbeer Al-GharaibehJan PurruckerAdam FlemingJohann SellnerAdina Turcu-StiolicaJohn MatsoukasAh-Mee ParkJuan Carlos López-GarcíaAjinkya S. SaseJudith DrenthenAlberto VerrottiKabirullah LutfyAlbrecht GuntherKally C. O’ReillyAlexander WeilKatarzyna HojanAlexandre González-RodríguezKathrine Bach SøndergaardAlexey BoykoKazuhiro P. IzawaAmad ZafarKeun-Yeong JeongAmy McTagueKunal DhimanAndré ToulouseKurt SvardsuddAndrea AlexandreLawrence S. HonigAndrea BartoliLiana DeheleanAndrew C. GallupLuigi F. IannoneAnna Jamroz-WiśniewskaMahesh KaushikAxel SteigerMarcello MocciaAyataka FujimotoMarcin ArciszewskiBarbara RemberkMarek KrzystanekBartłomiej Henryk NoszczykMargot ErnstBernard PossidenteMaria Giuseppina MianoBriana R. De MirandaMarsha PierceChenjie ShenMartin RegensburgerChun-An ChengMing ChenCristina DaiaMittal JasoliyaCyrus MotamedMonokesh Kumer SenDaniela CalinaMuck-Seler DoroteaDanuta Szkutnik-FiedlerMujeeb U. ShadDario SiniscalcoMuneaki MatsuoDaša StupicaNatalia MadetkoDavid KachlíkNathan K. EvansonDavide LugliettoNeepa J. PatelDiego CotellaNicola MontemurroDragos Catalin JianuNikolaos PitsikasDuncan HillNoriaki TomuraElena SavvateevaPamela McCombeElisa MaseroliPaolo ImmovilliEmanuel VamanuPatrycja KleczkowskaEmmanuel CognatPierre Eric JuifEnrico OpocherRaquel BarbosaEsteban SepúlvedaReem MasarwaFarid RahimiRenata BalnytėFederico Paolini Paolettiriantafyllos DidangelosFilippo M. SantorelliRita Maria Vittoria NobiliFrancesco AsciRoald Flesland HavreFrancesco CoreaRogério Leone BuchaimFrédéric GilbertRoman TrochimczukGaëtan LescaRomina RomanielloGeorgios TsivgoulisRosa De MiccoGerrit BrinkerRubén Darío Castro-TorresGhulam Md AshrafSamuel X. ShiGina FerrazzanoSergio FerrariGiorgio CalloviniShamik ShahGiovanni FerraraShitiz SriwastavaGonçalo S. DuarteSrihari GopalGoran KrstacicSteffen GrautoffHans-Peter HartungTaku SugiyamaHenry H. WooThorsten RudroffHongsun GuoTomasz FrączykHowan LeungTomohiko SadaokaIlona Rubi-FessenVaitsa Giannouli


